# A real-world retrospective analysis to evaluate the efficacy and safety of surufatinib in the treatment of advanced neuroendocrine neoplasms in China

**DOI:** 10.1530/EC-25-0543

**Published:** 2025-11-28

**Authors:** Shutong Liu, Yumeng Wang, Sen Hong, Lianru Zhang, Baorui Liu, Qin Liu

**Affiliations:** ^1^ Department of Oncology, Nanjing Drum Tower Hospital, Affiliated Hospital of Medical School, Nanjing University, Nanjing, China; ^2^ Clinical Cancer Institute of Nanjing University, Nanjing, China

**Keywords:** neuroendocrine neoplasms, surufatinib, neuroendocrine carcinomas, neuroendocrine tumors, real-world study

## Abstract

Surufatinib, an oral vascular immune kinase inhibitor targeting angiogenesis and immune escape, is approved in China for the treatment of unresectable locally advanced or metastatic neuroendocrine tumors (NETs). This real-world study aimed to evaluate its efficacy and safety in neuroendocrine neoplasms (NENs), including neuroendocrine carcinomas (NECs). From 2022 to 2024, 21 patients (12 with NET and 9 with NEC) at Nanjing Drum Tower Hospital received surufatinib-based therapy. The median follow-up period was 690 days. The primary endpoint, the disease control rate, was 81% (95% confidence interval (CI): 58.1–94.6), and the objective response rate (ORR) was 14% (95% CI: 3.0–36.3). The ORR was 8.3% (95% CI: 0.2–38.5) in NET and 44.4% (95% CI: 13.7–78.8) in NEC, with no significant difference by Fisher’s exact test (*P* = 0.119). The median progression-free survival (mPFS) was 9.7 months (95% CI: 2.5–15.9), while the median overall survival (mOS) had not been reached. For first-line treatment, the mPFS was 9.7 months (95% CI: 3.5–15.9) in NET patients and 15.1 months (95% CI: 3.8–26.5) in NEC patients; for second-line treatment, it was 6.7 months (95% CI: 0.9–12.4) in NET patients and 4.6 months (95% CI: 1.7–7.6) in NEC patients. Treatment-related adverse events (TRAEs) of grade ≥3 occurred in 28% of patients, and no treatment-related deaths were reported. Patients with TRAEs ≥1 (hazard ratio 0.349, *P* = 0.048) had longer PFS. This study supported the activity of surufatinib in advanced NENs; subgroup findings, including those for NEC, were exploratory and required confirmation.

## Introduction

The incidence of neuroendocrine neoplasms (NENs) was continuously increasing (1). In the United States, the incidence of NETs has increased more than six-fold over the last four decades. From 1973 to 2012, the incidence rate of gastroenteropancreatic (GEP) NECs rose consistently from 1.5 cases per 1,000,000 to 4.6 cases per 1,000,000 (2). In China, the overall age-standardized incidence rate (ASR) of NETs was 1.14 per 100,000. The prognosis for patients with metastatic or recurrent NENs is generally unfavorable, with limited treatment options available. For unresectable or metastatic diseases, treatment options include cytotoxic chemotherapy, somatostatin analogs, peptide receptor radionuclide therapy, everolimus, sunitinib, and surufatinib (3, 4). Surufatinib is the first domestically developed and innovative drug approved in China for the treatment of NETs. Surufatinib is an oral small-molecule vascular immune kinase inhibitor that targets vascular endothelial growth factor receptors (VEGFRs) 1/2/3, fibroblast growth factor receptor 1 (FGFR1), and colony-stimulating factor-1 receptor (CSF-1R). It works by inhibiting tumor angiogenesis and regulating tumor immune escape (5). Surufatinib has been approved in China for monotherapy in the treatment of unresectable locally advanced/metastatic NETs (6, 7, 8). Despite the high vascularization of NETs, few anti-angiogenic drugs have demonstrated efficacy in both pancreatic and extrapancreatic NETs in randomized controlled studies. Xu *et al.* were the first to use surufatinib in a phase Ib/II trial, exploring its potential in this context (9). Surufatinib was well tolerated and demonstrated responses in both pancreatic and extrapancreatic NETs. In two randomized controlled phase III clinical trials, surufatinib significantly improved progression-free survival (PFS) compared to placebo for both pancreatic and extrapancreatic NETs. The primary sites of extrapancreatic NETs were diverse, including gastrointestinal and extragastrointestinal tumor sources. Notably, prior therapies, including anti-angiogenic drugs, did not diminish the benefits of surufatinib on NETs. In addition, regardless of the NET type, the objective response rate (ORR) in the surufatinib group was significantly higher compared to placebo (6, 7). The patients in these two randomized controlled clinical studies were all at an advanced stage of the disease, suggesting that surufatinib could be a viable treatment option for unresectable locally advanced or metastatic tumors. However, research on surufatinib for patients with neuroendocrine carcinoma (NEC) remains limited (10). Zhang *et al.* demonstrated that the combination of surufatinib and toripalimab exhibited anti-tumor activity and tolerable safety in 21 patients with previously treated advanced NECs. The mPFS was 4.1 months (1.5–5.5), and the median overall survival (OS) was 10.9 months (9.1–14.6) (11). Currently, large-scale, randomized controlled clinical trials are lacking to substantiate the efficacy of surufatinib in the treatment of patients with NEC.

Exploration of biomarkers was conducted in these clinical trials. No significant increase in bFGF was observed compared to baseline. Previous studies indicated that an increase in bFGF was associated with disease progression after treatment with anti-angiogenic drugs, suggesting that FGF activation may contribute to acquired resistance to other anti-angiogenic therapies. However, this mechanism was not identified for surufatinib. In addition, an increase in tumor-associated macrophages (TAMs) was found to be linked to accelerated disease progression in advanced NETs. TAMs interact with NET cells by activating EGFR, CSF-1R, and the WNT signaling pathway. Surufatinib’s inhibition of CSF-1, VEGFR, and FGF signals may help explain its effectiveness in controlling disease progression in advanced NETs.

Although surufatinib has demonstrated efficacy in clinical trials for advanced NENs, its actual benefits in real-world settings remain unclear due to the heterogeneity of NENs and variations in medication histories. Therefore, understanding its effectiveness and safety in the diverse Chinese population has become increasingly important. This study aims to retrospectively evaluate the efficacy and safety of surufatinib in patients with NENs in China, providing insights to guide treatment selection and enhance patient outcomes in real-world performance.

## Materials and methods

**Figure 1:**
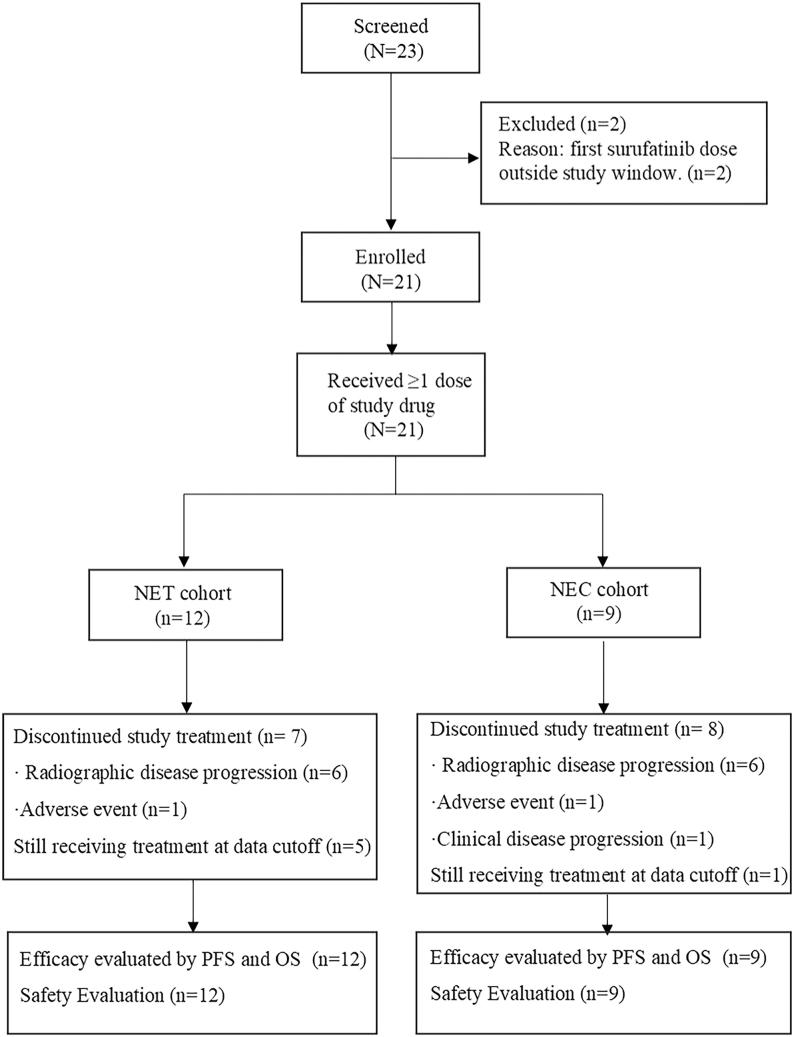
Flow chart. NEC, neuroendocrine carcinoma; NET, neuroendocrine tumor; PFS, progression-free survival; OS, overall survival.

We conducted a single-center retrospective cohort study at Nanjing Drum Tower Hospital. By querying the hospital’s pharmacy database and cross-checking the electronic medical record system, we included all consecutive adult patients who were diagnosed with unresectable, locally advanced, or metastatic NENs and had received at least one dose of surufatinib between January 1, 2022, and August 30, 2024. Eligibility required histological confirmation of NET or NEC, radiological evidence of unresectable, locally advanced, or metastatic disease, and receipt of the first surufatinib dose within the study window; evaluability furthermore required baseline cross-sectional imaging with CT or MRI obtained within 28 days before the first dose. Patients were excluded only when these prespecified conditions were not met, including a first dose outside the study window, absence of baseline imaging, or withdrawal before the first dose, and no physician-discretionary selection was applied beyond these criteria. The screening cascade was shown in Fig. 1. The observation period began at the first administration of surufatinib and continued until radiographic progression, death, loss to follow-up, or the data cut-off. Tumor response and progression were assessed by two radiologists according to RECIST v1.1 at approximately 6–9 week intervals. Any discrepancies in the assessment results were resolved through consensus discussion. Concomitant anticancer therapies, line of therapy, and treatment duration were abstracted from the record for descriptive reporting. All consecutive eligible patients were included in the analyses, and no imputation was performed for missing efficacy data. The study was approved by the Ethics Committee of Nanjing Drum Tower Hospital (2024-635-01) and conducted in accordance with the Declaration of Helsinki. Consent was obtained from each patient or subject after full explanation of the purpose and nature of all procedures used.

### Treatment

In this study, all patients received targeted therapy with surufatinib, with the daily dosage adjusted according to their disease conditions and performance status, ranging from 200 to 300 mg. For patients with NEC, combination treatment modalities, including chemotherapy, immunotherapy, and radiotherapy, were commonly employed. In contrast, patients with NET underwent comprehensive treatment strategies that involved either the single-agent use of surufatinib or its combination with somatostatin analogs. Treatment with surufatinib continued until disease progression, intolerable adverse events occurred, or the patient passed away.

### Efficacy and safety evaluation

During the treatment, all patients underwent imaging evaluations using computed tomography (CT) and/or magnetic resonance imaging (MRI). Treatment responses were classified according to the Response Evaluation Criteria in Solid Tumors (RECIST) version 1.1 as complete response (CR), partial response (PR), stable disease (SD), and progressive disease (PD). The objective response rate (ORR) was calculated by summing the proportions of patients with CR and PR, while the disease control rate (DCR) was determined by the proportion of patients achieving CR, PR, and SD. PFS was measured from the date of the first surufatinib dose to radiographic progression or death from any cause, whichever occurred first. OS was measured from the date of the first surufatinib dose to death from any cause; patients alive at last contact were censored at that date. The primary outcome was DCR, with secondary outcomes including ORR, PFS, and OS. Treatment-related adverse events (TRAEs) were collected from medical records and classified according to the Common Terminology Criteria for Adverse Events (CTCAE) version 5.0.

### Statistical analysis

We summarized categorical variables as counts and percentages and continuous variables as medians with interquartile ranges. Objective response rate and disease control rate were reported with two-sided 95% confidence intervals (CI) using the exact Clopper–Pearson method, and group differences in proportions were tested with two-sided Fisher’s exact test. Progression-free survival and OS were estimated by the Kaplan–Meier method from the date of the first surufatinib dose; medians and time-point survival probabilities were accompanied by two-sided 95% CI, and groups were compared using the log-rank test. Hazard ratios (HRs) with 95% CI were obtained from Cox proportional hazards models, and the proportional hazards assumption was assessed using standard graphical diagnostics and time-dependent covariate tests. In view of the limited number of events, multivariable modeling was constrained and interpreted as exploratory, and we reported adjusted effects together with the overall likelihood-ratio test for model coefficients. All tests were two-sided with a significance level of 0.05; no adjustment for multiplicity was applied, and missing efficacy data were not imputed. Analyses were performed with IBM SPSS Statistics version 26.0.

## Results

### Patient characteristics

From January 1, 2022, to August 30, 2024, a total of 21 patients received comprehensive treatment, including surufatinib, at our institution. All patients were diagnosed with locally advanced, unresectable, or metastatic NEC/NET and underwent imaging evaluations. The baseline characteristics of these patients were summarized in Table 1. The median age of participants was 63 years, with a range of 42–82 years, comprising 15 males and 6 females. Among the 21 patients, 16 (76%) had never smoked, while 5 (24%) were smokers. The Eastern Cooperative Oncology Group (ECOG) performance status indicated that 9 patients (43%) had a score of 0 and 12 patients (57%) had a score of 1. Diagnostically, 9 patients (43%) had NEC, and 12 patients (57%) had NET. Regarding metastatic disease, four patients (19%) had oligometastatic lesions, whereas 17 (81%) had extensive metastasis. The liver was the most common metastatic site, affecting 15 patients (72%), with all patients having liver metastasis among those with multiple metastatic lesions. Tumor characteristics showed that 13 patients (62%) had Ki-67 expression <55%, while 7 patients (34%) had Ki-67 expression ≥55%. In terms of chromogranin A (CgA) expression, 5 patients (24%) had negative results, and 14 (67%) had positive results. Treatment history revealed that 8 patients (38%) had received chemotherapy, 7 (33%) had undergone immunotherapy, and 13 (62%) had undergone surgery. Notably, 15 patients (72%) received surufatinib as first-line treatment, including 6 diagnosed with NEC and 9 with NET.

**Table 1:** Patient baseline demographic and baseline disease characteristics.

Characteristics	All patients *n* = 21
Age, years	
Median (range)	63 (42–82)
<65 years old, *n* (%)	11 (52)
≥65 years old, *n* (%)	10 (48)
Sex, *n* (%)	
Female	6 (29)
Male	15 (71)
Smoking status, *n* (%)	
Never	16 (76)
Former/current	5 (24)
ECOG PS, *n* (%)	
0	9 (43)
1	12 (57)
Tumor stage at screening, *n* (%)	
IV	21 (100)
WHO classification, *n* (%)	
NET	12 (57)
G1	1 (4)
G2	9 (43)
G3	2 (10)
NEC	9 (43)
Primary tumor location, *n* (%)	
Extra-pancreatic	16 (76)
Pancreas	5 (24)
Metastasis	
Oligometastasis, *n* (%)	17 (81)
Extensive metastasis, *n* (%)	4 (19)
Ki-67, *n* (%)	
<55%	13 (62)
≥55%	7 (34)
NA	1 (4)
CgA, *n* (%)	
(−)	5 (24)
(+)	6 (29)
≥ (++)	8 (38)
NA	2 (9)
Combined with immunotherapy, *n* (%)	
Yes	7 (33)
Surgery, *n* (%)	
Yes	13 (62)
No	8 (38)
Time from diagnosis to oral surufatinib, *n* (%)	
<6 months	12 (57)
≥6 months	9 (43)
Systemic therapy, *n* (%)	
Yes	6 (29)
No	15 (71)
Chemotherapy, *n* (%)	
Yes	8 (38)
No	13 (62)
Lines of prior anti-tumor therapies, *n* (%)	
0	15 (72)
1	5 (24)
≥2	1 (4)

NEC, neuroendocrine carcinoma; NET, neuroendocrine tumor; ECOG, Eastern Cooperative Oncology Group.

### Efficacy

**Figure 2:**
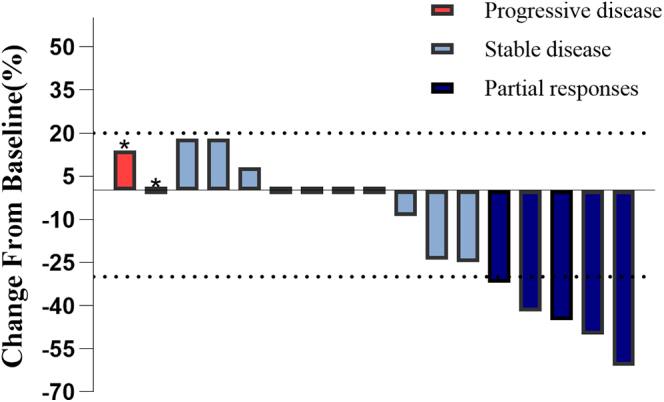
A waterfall plot of tumor volume change when the optimal efficacy was achieved. The dotted lines of ‘+20%’ and ‘−30%’ indicate the thresholds for progressive disease and partial responses. ‘*’ indicates that the patient is judged to have progressed due to the presence of a new lesion.

**Figure 3:**
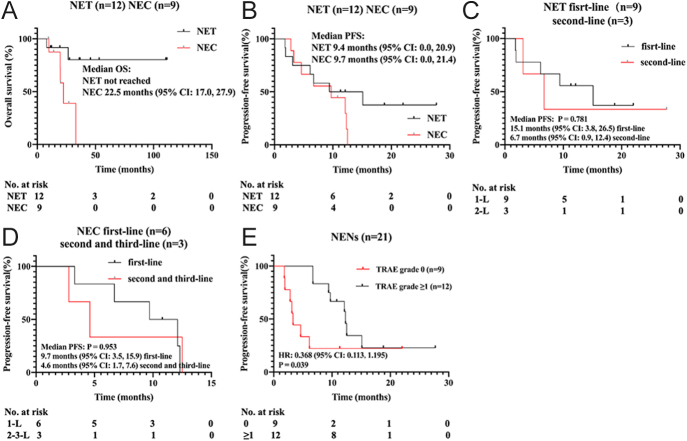
Kaplan–Meier estimates of PFS by tumor cohort. Tumor response was assessed by the investigator using RECIST v1.1. NENs, neuroendocrine neoplasms; NEC, neuroendocrine carcinoma; NET, neuroendocrine tumor; PFS, progression-free survival; TRAE, treatment-related adverse event.

Tumor volume changes at the time of achieving the best response were assessed in 17 patients with available imaging materials, as shown in Fig. 2. According to RECIST version 1.1, 5 patients were classified as having a partial response (PR), 12 as having stable disease (SD), and 4 as having progressive disease (PD) (Fig. 2, Table 2). The ORR of all patients was 23.8% (95% CI: 0.082, 0.472), while the DCR was 81% (95% CI: 0.581, 0.946). The ORR was 8.3% (95% CI: 0.002, 0.385) in the NET cohort and 44.4% (95% CI: 0.137, 0.788) in the NEC cohort. The DCR was 83% (95% CI: 0.516, 0.979) in the NET cohort and 77.8% (95% CI: 0.400, 0.972) in the NEC cohort (Table 2). The median follow-up time for patients was 690 days. Kaplan-Meier survival analysis indicated that, as of January 27, 2025, the mPFS of the enrolled patients was 9.7 months (95% CI: 2.5, 16.8), and the mOS had not been reached. In subgroup analyses, the median OS was 22.5 months (95% CI: 17.0, 27.9) for the NEC group, and the NET group did not reach the median OS (Fig. 3A). The median PFS was 9.4 months (95% CI: 0.0, 20.9) for the NET group and 9.7 months (95% CI: 0.0, 21.4) for the NEC group (Fig. 3B). Within the NET group, nine patients received surufatinib as first-line treatment, resulting in a mPFS of 15.1 months (95% CI: 3.8, 26.5) (Fig. 3C). In addition, three patients in the NET group who received surufatinib as second-line treatment had a PFS of 6.7 months (95% CI: 0.9, 12.4) (Fig. 3C). In the NEC group, six patients received surufatinib as first-line treatment, with a median PFS of 9.7 months (95% CI: 3.5, 15.9) (Fig. 3D), while three patients who received it as second-line treatment had a median PFS of 4.6 months (95% CI: 1.7, 7.6) (Fig. 3D).

**Table 2:** Treatment response with targeted agent surufatinib.

Treatment response	NET		NEC		*P* value
	*n* (%)	95% CI	*n* (%)	95% CI	
Best response					
PR	1 (8.3)		4 (44.5)		
SD	9 (75.0)		3 (33.3)		
PD	2 (16.7)		2 (22.2)		
ORR	8.3	0.002–0.385	44.4	0.137–0.788	0.119
DCR	83.3	0.516–0.979	77.8	0.400–0.972	1.000

NEC, neuroendocrine carcinoma; NET, neuroendocrine tumor; PR, partial response; SD, stable disease; PD, progressive disease; ORR, objective response rate; DCR, disease control rate. The 95% CI of ORR and DCR are based on the Clopper-Pearson method.

Univariate Cox regression analysis of OS identified one variable with a significant association and several variables without statistically significant associations. Diagnosis of NET was associated with longer OS (HR 0.171, *P* = 0.041). Other variables with HR below 1, including age ≥65 years (HR 0.700, *P* = 0.643), female sex (HR 0.533, *P* = 0.562), liver metastasis (HR 0.531, *P* = 0.472), prior or concomitant surgery (HR 0.178, *P* = 0.050), and side effects graded ≥1 (HR 0.688, *P* = 0.626), were not significantly associated with OS. Variables with numerically higher hazards included an ECOG score of 1 (HR 7.021, *P* = 0.075), past or current smoking (HR 1.724, *P* = 0.477), primary tumor location outside the pancreas (HR 1.880, *P* = 0.561), extensive metastasis (HR 1.556, *P* = 0.599), Ki-67 ≥55% (HR 1.682, *P* = 0.555), and receipt of immunotherapy (HR 5.440, *P* = 0.046), systemic anti-tumor treatment (HR 1.102, *P* = 0.901), chemotherapy (HR 5.290, *P* = 0.050), and hypertension (HR 11.968, *P* = 0.026), as detailed in Table 3.

**Table 3:** Cox regression analysis of OS.

Factor	Univariate		Multivariate	
	HR (95% CI)	*P* value	HR (95% CI)	*P* value
Age				
<65 years old	Reference			
≥65 years old	0.700 (0.156–3.153)	0.643		
Sex				
Male	Reference			
Female	0.533 (0.064–4.455)	0.562		
Smoking status				
Never	Reference			
Former/current	1.724 (0.385–7.723)	0.477		
ECOG				
0	Reference		Reference	
1	7.021 (0.822–59.939)	0.075	2.146 (0.138–33.333)	0.585
WHO classification				
NEC	Reference		Reference	
NET	0.041 (0.031–0.934)	0.041	2.702 (0.041–177.463)	0.642
Primary tumor location				
Pancreas	Reference			
Extra-pancreatic	1.880 (0.334–15.744)	0.561		
Metastasis				
Oligometastasis	Reference			
Extensive metastasis	1.599 (0.299–8.089)	0.599		
Liver metastasis				
No	Reference			
Yes	0.531 (0.095–2.980)	0.472		
Ki-67				
<55%	Reference			
≥55%	1.682 (0.300–9.446)	0.555		
CgA				
< (++)	Reference			
≥ (++)	0.185 (0.000–154.836)	0.135		
Combined with immunotherapy				
No	Reference		Reference	
Yes	5.440 (1.034–28.629)	0.046	5.559 (0.198–156.275)	0.314
Surgery				
No	Reference		Reference	
Yes	0.178 (0.032–1.000)	0.050	0.523 (0.054–5.083)	0.577
Systemic therapy				
No	Reference			
Yes	1.102 (0.241–5.043)	0.901		
Chemotherapy				
No	Reference		Reference	
Yes	5.290 (1.003–27.893)	0.050	4.529 (0.114–180.210)	0.422
Any-grade TRAE ≥1				
No	Reference			
Yes	0.688 (0.153–3.101)	0.626		
Hypertension				
No	Reference		Reference	
Yes	11.968 (1.343–106.674)	0.026	13.428 (0.378–477.214)	0.154

NEC, neuroendocrine carcinoma; NET, neuroendocrine tumor; ECOG, Eastern Cooperative Oncology Group; OS, overall survival; TRAE, treatment-related adverse event. Cox estimates are presented as hazard ratios with two-sided 95% confidence intervals. Analyses are exploratory due to the limited number of events; variables with confidence intervals crossing 1 are not statistically significant.

Univariate Cox analysis for PFS identified one significant association and several non-significant observations (Table 4). Patients with side-effect grade ≥1 had longer PFS (HR 0.349, *P* = 0.048). Variables with HR below 1, including diagnosis of NET (HR 0.522, *P* = 0.235) and extensive metastasis (HR 0.571, *P* = 0.403), were not significantly associated with PFS. Variables with numerically higher hazards – including primary tumor outside the pancreas (HR 2.037, *P* = 0.351), concomitant immunotherapy (HR 1.275, *P* = 0.648), time from diagnosis to surufatinib ≥6 months (HR 1.291, *P* = 0.634), prior systemic anti-tumor therapy (HR 1.428, *P* = 0.520), chemotherapy (HR 2.635, *P* = 0.076), and hypertension (HR 1.457, *P* = 0.496) – also did not reach statistical significance. Kaplan–Meier estimates were consistent: median PFS was 12.3 months (95% CI: 11.8–12.9) in patients with side-effect grade ≥1 and 3.3 months (95% CI: 2.7–3.9) in those with grade 0, with a log-rank *P* value of 0.039 (Fig. 3E).

**Table 4:** Cox regression analysis of PFS.

Factor	HR	Univariate	*P* value
		95% CI	
WHO classification			
NEC		Reference	
NET	0.522	0.179–1.525	0.235
Primary tumor location			
Pancreas		Reference	
Extra-pancreatic	2.037	0.457–9.081	0.351
Metastasis			
Oligometastasis		Reference	
Extensive metastasis	0.571	0.153–2.126	0.403
Liver metastasis			
No		Reference	
Yes	0.857	0.250–2.934	0.805
Ki-67			
<55%		Reference	
≥55%	0.905	0.302–2.718	0.859
CgA			
< (++)		Reference	
≥ (++)	1.198	0.401–3.579	0.600
Combined with immunotherapy			
No		Reference	
Yes	1.275	0.450–3.617	0.648
Surgery			
No		Reference	
Yes	0.970	0.343–2.741	0.954
Time from diagnosis to oral surufatinib			
<6 months		Reference	
≥6 months	1.291	0.452–3.687	0.634
Systemic therapy			
No		Reference	
Yes	1.428	0.482–4.228	0.520
Chemotherapy			
No		Reference	
Yes	2.635	0.903–7.684	0.076
Any-grade TRAE ≥1			
No		Reference	
Yes	0.349	0.122–0.993	0.048
Hypertension			
No		Reference	
Yes	1.457	0.493–4.304	0.496

NEC, neuroendocrine carcinoma; NET, neuroendocrine tumor; PFS, progression-free survival. TRAE, treatment-related adverse event. Cox estimates are presented as hazard ratios with two-sided 95% confidence intervals. Analyses are exploratory; CIs crossing 1 indicate non-significance.

### Safety

All TRAEs were detailed in Table 5. In general, most patients experienced at least one TRAE, with the majority being grade 1 or 2. Six patients encountered serious TRAEs (≥ grade 3), including one patient with grade 3 diarrhea, one with grade 3 leukopenia, one with grade 4 leukopenia, one with grade 4 thrombocytopenia, one patient with both grade 4 leukopenia and grade 4 thrombocytopenia, and another patient with grade 3 anemia and grade 3 proteinuria. The most common adverse reactions during treatment were proteinuria (6/21, 29%), followed by hypertension (4/21, 19%), increased urea nitrogen (4/21, 19%), increased creatinine (3/21, 14%), hyperuricemia (3/21, 14%), hypokalemia (3/21, 14%), hypertriglyceridemia (3/21, 14%), increased aspartate aminotransferase (3/21, 14%), and hypoalbuminemia (3/21, 14%). The most common hematological events included leukopenia (4/21, 19%), anemia (2/21, 9%), and thrombocytopenia (2/21, 9%). During treatment with surufatinib, two patients discontinued due to grade 4 thrombocytopenia and severe grade 3 diarrhea, respectively. Importantly, there were no treatment-related deaths reported during the study.

**Table 5:** Summary of study treatment-related adverse events by cohort.

Summary of TRAEs, *n* (%)	NET	NEC	Total
	*n* = 12	*n* = 9	*n* = 21
Any TRAEs	8 (67)	6 (67)	14 (67)
Grade ≥3	4 (33)	5 (56)	6 (29)
Surufatinib-related adverse events	7 (58)	6 (67)	13 (62)
Grade ≥3	3 (25)	5 (56)	6 (29)
Leading to discontinuation of surufatinib	2 (17)	1 (11)	3 (14)

TRAEs by surufatinib, *n* (%)	NET		NEC			Total
	*n* = 12		*n* = 9			*n* = 21
	Grade 1–2	Grade 3	Grade 1–2	Grade 3	Grade 4	
Proteinuria	1 (8)	1 (8)	4 (44.4)	0	0	6 (29)
White blood cell decreased	0	0	1 (11)	1 (11)	2 (22)	4 (19)
Hypertension	3 (25)	0	1 (11)	0	0	4 (19)
Urea nitrogen increase	2 (17)	0	2 (22)	0	0	4 (19)
Hyperuricemia	0	0	3 (33)	0	0	3 (14)
Creatinine increased	1 (8)	0	2 (22)	0	0	3 (14)
Hypokalemia	2 (17)	0	1 (11)	0	0	3 (14)
Hypertriglyceridemia	1 (8)	0	2 (22)	0	0	3 (14)
Aspartate aminotransferase increased	0	0	3 (33)	0	0	3 (14)
Edema limbs	1 (8)	1 (8)	1 (11)	0	0	3 (14)
Hypoalbuminemia	2 (17)	0	1 (11)	0	0	3 (14)
Hypothyroidism	2 (17)	0	0	0	0	2 (9)
Anemia	0	1 (8)	1 (11)	0	0	2 (9)
Platelet count decreased	0	0	0	0	2 (22)	2 (9)
Hypertriglyceridemia	1 (8)	0	1 (11)	0	0	2 (9)
Diarrhea	1 (8)	1 (8)	0	0	0	2 (9)
Blood bilirubin increased	0	0	2 (22)	0	0	2 (9)
Gastroesophageal reflux disease	0	0	1 (11)	0	0	1 (5)
Upper gastrointestinal hemorrhage	1 (8)	0	0	0	0	1 (5)
Hematuria	1 (8)	0	0	0	0	1 (5)
Malaise	1 (8)	0	0	0	0	1 (5)
Palpitation	1 (8)	0	0	0	0	1 (5)
Cholesterol high	0	0	1 (11)	0	0	1 (5)
Nausea	1 (8)	0				

TRAEs, treatment-related adverse events; NEC, neuroendocrine carcinoma; NET, neuroendocrine tumor.

## Discussion

In this real-world retrospective observational study, surufatinib demonstrated anti-tumor activity and an acceptable safety profile in patients with unresectable, locally advanced, or metastatic NENs.

The median PFS for all patients was 9.7 months (95% CI: 2.5, 16.8), while the median OS was not reached. In the NET group, which included 12 patients, those receiving surufatinib as first-line treatment had a median PFS of 15.1 months (95% CI: 3.8, 26.5), compared to a median PFS of 6.7 months (95% CI: 0.9, 12.4) for those treated in the second line. Previous evidence of surufatinib in advanced NET demonstrated that the PFS ranged from 7.4 to 13.8 months (6, 7), which was similar to our results. There were a total of nine patients in the NEC group. In this cohort, those who received surufatinib as the first-line treatment had a median PFS of 9.7 months (95% CI: 3.5, 15.9), while those treated in the second line had a median PFS of 4.6 months (95% CI: 1.7, 7.6). In addition, the median PFS with surufatinib plus toripalimab for second-line treatment of NEC was 4.1 months, as reported in a multicenter, single-arm phase II study (11). In our study, earlier initiation and first-line use of surufatinib were associated with numerically longer PFS, but these findings are hypothesis-generating. Given small subgroups and the absence of prespecified, adequately powered comparisons, a causal benefit of earlier use cannot be inferred and requires confirmation in larger prospective studies.

Some histopathological factors, such as grading, classification, staging, and Ki-67 index, were identified as related to mOS and mPFS, which had already been validated by numerous studies (12, 13). In previous studies, the liver was the most common site of metastasis and a common influencing factor (1, 6, 7). Liver metastasis may influence OS in NENs and was an instructive prognostic factor, which had been validated by previous studies (14, 15). In a subgroup analysis of a phase Ib/II clinical study evaluating the regimen of surufatinib combined with camrelizumab, albumin-bound paclitaxel, and S-1 as first-line treatment for metastatic pancreatic ductal adenocarcinoma (mPDAC), the ORR for pancreatic cancer patients with liver metastases who received the combination treatment was significantly higher than that of patients without liver metastases (90.0 vs 20.0%, *P* = 0.0017). Moreover, patients without liver metastases had a significantly longer mPFS compared to those with liver metastases (9.4 vs 5.7 months, *P* = 0.0053). This study suggests that surufatinib may provide greater benefits to patients with liver metastases (16). In our analysis, liver metastasis status was not significantly associated with PFS (HR = 0.857, *P*-value = 0.805). This association did not reach statistical significance, and this result is likely attributed to the small sample size and the limited number of key events (e.g., disease progression). Therefore, any observed numerical differences should be regarded as exploratory findings.

Compared to NET, poorly differentiated NEC exhibits a higher degree of malignancy. In our cohort, NEC showed numerically shorter OS and PFS than NET, but subgroup comparisons were underpowered and are presented as exploratory rather than definitive. Tumor metastasis can occur early in the disease, and traditional chemotherapy has limited efficacy for NEC. As a result, combination therapy has become a common approach in tumor treatment, incorporating agents such as somatostatin analogs (SSAs), chemotherapy, and immune checkpoint inhibitors (ICIs) to meet practical treatment needs. The finding that combined immunotherapy was a negative prognostic factor for OS may be attributed to the timing of treatments. Most NEC patients received combined immunotherapy as second-line treatment, while others were treated with chemotherapy combined with surufatinib, etoposide, and cisplatin in the first line. This difference in treatment timing complicates survival comparisons, limiting their clinical significance. A study by Wenfeng Fang *et al.* explored a novel quadruple-drug regimen of surufatinib, toripalimab, etoposide, and cisplatin as first-line treatment for extensive-stage small-cell lung cancer (ES-SCLC). This regimen combines anti-angiogenic and anti-PD-1 therapies with chemotherapy, targeting tumors through multiple biological mechanisms. In this study, the ORR of the patients was 97.1% (34/35), the DCR and the tumor shrinkage rate were both 100% (35/35). The PFS was 6.9 months (95% CI: 4.6–9.2), and the OS was 21.1 months (95% CI: 12.1–30.1). This innovative combination fills a research gap and offers a new treatment option (17). In our study, most patients with NEC had carcinomas originating from the digestive system, with only two patients having primary tumors in the ureter. Surufatinib combined with immunotherapy demonstrated a promising therapeutic effect, with an ORR of 28.6% and a DCR of 71.4%. Among the seven patients who received combination immunotherapy, one was treated in the first line, four in the second line, and two in the third line. One female patient treated with surufatinib and immunotherapy in the first line had extensive tumor metastases at diagnosis. Her condition deteriorated rapidly, resulting in a PFS of only 3.3 months and an OS of 10.0 months. A male patient receiving combination immunotherapy in the third line had poor performance status and rapid disease progression, achieving a PFS of just 2.8 months and dying 4 months after progression. These cases suggest that the overall treatment effects of surufatinib combined with immunotherapy may have been suboptimal due to the advanced stage of the disease or the late-line use of the combination therapy.

In general, the safety profile of surufatinib was similar to the reports of randomized controlled trials (6, 7). Interestingly, patients with grade ≥1 side effects had a better prognosis and longer PFS compared to those without side effects. The median PFS durations were 12.3 months for patients with grade ≥1 side effects and 3.3 months for those without side effects, showing statistically significant differences. Clinical trials also indicated that the presence of TRAEs was significantly associated with longer PFS (18). Therefore, the presence of TRAEs should be accorded significant clinical consideration, and it is recommended to assess the baseline status of patients with drug toxicity to better control and manage side effects. Hypertension is one of the common adverse reactions of surufatinib. Our safety analysis also demonstrated that in three NET patients, their blood pressure changed from normal to grade 1 hypertension after taking surufatinib for a period of time. One NEC patient, who had pre-existing grade 1 hypertension, progressed to grade 2 hypertension after taking surufatinib for 6 months. The blood pressure of the remaining patients with a history of hypertension remained relatively stable during the period of surufatinib treatment. The OS of patients with a history of hypertension was less favorable than that of patients without a history of hypertension (HR 11.968, *P* = 0.026). However, the results of the multivariate analysis were not statistically significant. Meanwhile, we analyzed the relationship between post-treatment blood-pressure increase and PFS (HR 0.499, *P* = 0.363). This comparison was not statistically significant, and any apparent numerical difference should be regarded as exploratory. This suggests that when using surufatinib, we should pay more attention to changes in patients’ blood pressure, manage it effectively, and ensure that patients’ prognoses are optimized while improving efficacy.

In this real-world study, we complement previous research reports on surufatinib by focusing on describing the clinical treatment practices for NETs and NECs at a tertiary first-class hospital in China. These practices specifically include the use of surufatinib in first-line and later-line treatments, monotherapy and combination therapy regimens, as well as routine dose adjustments during treatment. Beyond verifying the efficacy of surufatinib in unselected real-world clinical settings, we also present two exploratory observations that may provide references for the development of future research hypotheses: first, there is an association between TRAEs of any grade and longer PFS in routine clinical practice; second, we provide descriptive analyses of PFS and ORR data in NEC patients treated with surufatinib-based mixed regimens.

These observations are limited by insufficient statistical power, and the study design itself is not intended for causal inference. However, they refine the range of expected benefits of surufatinib in routine clinical practice and identify patient subgroups and study design elements that merit verification in prospective studies.

Our study has several limitations. This single-center retrospective design without a comparator precludes causal inference and comparative-effectiveness conclusions. Despite consecutive inclusion within a predefined window and prespecified eligibility and evaluability criteria, residual selection and information biases are unavoidable in routine clinical datasets. The small sample and limited number of events render subgroup analyses – NET versus NEC, first-line versus later lines, and monotherapy versus combination – underpowered and strictly exploratory. Collectively, these data are hypothesis-generating and require confirmation in larger, prospective, multicenter studies with appropriate comparators.

## Declaration of interest

The authors declare that there are no conflicts of interest regarding the publication of this article.

## Funding

This study was funded by Jiangsu Provincial Medical Key Discipline (NO. ZDXK202233).

## Data availability

The dataset used and analyzed during the current study is available from the corresponding author on reasonable request.
